# CircRNA WHSC1 promotes non‐small cell lung cancer progression via sponging microRNA‐296‐3p and up‐regulating expression of AKT serine/threonine kinase 3

**DOI:** 10.1002/jcla.23865

**Published:** 2021-07-27

**Authors:** Fengfeng Shi, Qin Yang, Dongdong Shen, Jianwei Chen

**Affiliations:** ^1^ Department of Thoracic Surgery Cixi People's Hospital Cixi City China; ^2^ Department of Respiratory Medicine Cixi People's Hospital Cixi City China

**Keywords:** AKT3, circWHSC1, miR‐296‐3p, non‐small cell lung cancer

## Abstract

**Background:**

Lung cancer is the most commonly diagnosed cancer and leading cause of cancer death, with 80%–85% of non‐small cell lung cancer (NSCLC). Circular RNAs (circRNAs) have been shown to be promising early diagnostic and therapeutic molecular biomarkers for NSCLC. However, biological role and regulatory mechanism of circRNA WHSC1 (circWHSC1) in NSCLC are unknown. Therefore, we aim to explore the function and mechanism of circWHSC1 in NSCLC oncogenesis and progression.

**Methods:**

qRT‐PCR was used for circWHSC1 level evaluation; Kaplan‐Meier was used for survival analysis; bioinformatics, dual‐luciferase activity, and RNA pull‐down were used for evaluating competing endogenous RNA (ceRNA) network; cell viability, colony formation, apoptosis, migration, and invasion were used for cell function analysis; function gain and loss with rescue experiments were used for exploring mechanism of circWHSC1 in NSCLC development.

**Results:**

Significantly up‐regulated circWHSC1 and down‐regulated microRNA‐296‐3p (miR‐296‐3p) were identified in NSCLC tissues and cells. Up‐regulated circWHSC1 was associated with poor prognosis in NSCLC patients. MiR‐296‐3p was sponged by circWHSC1, and AKT serine/threonine kinase 3 (AKT3) was target of miR‐296‐3p; meanwhile, miR‐296‐3p over‐expression significantly down‐regulated AKT3 expression, and co‐transfecting anti‐miR‐296‐3p rescued circWHSC1 silence caused AKT3 down‐regulation. CircWHSC1 silence significantly inhibited colony formation, viability, invasion, and migration, while increased NSCLC cell apoptosis, which were partially rescued by anti‐miR‐296‐3p.

**Conclusion:**

CircWHSC1 is an independent indicator of poor prognosis in NSCLC patients, and functions as a ceRNA of miR‐296‐3p to up‐regulate AKT3, consequently promotes NSCLC cell growth and metastasis. Targeting circWHSC1 might be a prospective strategy for diagnosis, therapeutics, and prognosis of NSCLC.

## Graphical abstract

However, miR‐296‐3p over‐expression significantly down‐regulated AKT3 expression, and co‐transfecting anti‐miR‐296‐3p rescued circWHSC1 silence caused down‐regulation of AKT3. Therefore, circWHSC1 is an independent indicator of poor prognosis in NSCLC patients, and functions as a competing endogenous RNA (ceRNA) of miR‐296‐3p to up‐regulate AKT3, consequently promotes NSCLC cell growth and metastasis.

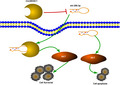

## 1 INTRODUCTION

As the second most commonly diagnosed cancer and the leading cause of cancer death, lung cancer accounts for about one in 10 (11.4%) diagnosed malignancies and one in 5 (18.0%) deaths, with an estimated 2.2 million new patients and 1.8 million deaths in 2020.^1^ Non‐small cell lung cancer (NSCLC) is the most common pathological type, accounting for 80% to 85%, of lung cancer.^2^, ^3^, ^4^ Even with recent breakthroughs in early diagnosis and treatment, the clinical outcome of NSCLC patients remains poor with a 5‐year overall survival rate less than 20%; moreover, the incidence of NSCLC is still rising.^5^, ^6^ Consequently, it is crucial to further discover the underlying mechanisms contributing to NSCLC pathogenesis and metastasis, which will provide prospective biomarkers for exploring novel and more effective molecularly targeted therapies.

Since first discovered in 1976, various circular RNAs (circRNAs) have been discovered, which are produced by backsplicing and more resistant to exonuclease.^7^, ^8^ CircRNAs play essential roles in regulating many different physiological and pathological processes, including carcinogenesis and development of NSCLC ^9^, ^10^; meanwhile, due to their stability in diverse body fluids, circRNAs may serve as promising early diagnostic/prognostic biomarkers and potential targeted therapeutic targets for NSCLC and have become the focus of research on NSCLC.^9^, ^10^, ^11^, ^12^, ^13^ Moreover, circRNAs predominantly involve in transcriptional gene regulation by acting as sponges of miRNAs.^14^, ^15^, ^16^, ^17^

CircWHSC1 has been reported to serve as an oncogene in promoting the development of hepatocellular carcinoma,^18^ endometrial cancer,^19^ and ovarian cancer.^20^ So far, the expression profile, biological function, and mechanism of circWHSC1 in NSCLC have not been studied.

MiR‐296‐3p has been reported to suppress the viability, migration, and invasion of NSCLC cells.^21^, ^22^

Here, we investigated the circWHSC1 expression profile in NSCLC patient tissues and cells and found that circWHSC1 was significantly increased in NSCLC tissues and related to the prognosis of NSCLC patients. Furthermore, we found that circWHSC1 may serve as a sponge of miR‐296‐3p to increase AKT3 expression and, subsequently, promote NSCLC development. Thus, up‐regulated circWHSC1 may function as a biomarker for predicting prognosis and promising therapeutic target in NSCLC patients.

## 2 MATERIALS AND METHODS

### 2.1 Patient specimens

Cancerous and paired paracancerous pulmonary specimens were harvested from 70 diagnosed NSCLC patients when subjected to surgical treatment in our hospital, which were frozen in liquid nitrogen immediately.

### 2.2 Reagents

Dulbecco's Modified Eagle Medium (DMEM) and fetal bovine serum (FBS) (Gibco); 24‐well transwells with 8.0 μm pore (Corning Costar); Dual‐Luciferase Reporter Assay Kit (Yeasen); Lipofectamine 2000, SuperSignal West Dura Extended Duration Substrate, Streptavidin agarose magnetic beads and Revert Aid First Strand cDNA Synthesis Kit (Thermo Fisher); psiCHECK™‐2 vector (Promega); CCK‐8 assay solution and Annexin V/FITC Apoptosis Detection Kit (Dojindo Corp); Gene Mutation Kit and SYBR Green PCR Master Mix (Takara); Matrigel (BD.); RIPA lysis buffer (Beyotime); antibodies (Santa Cruz).

### 2.3 Cells and culture

Human NSCLC (CALU3, CALU6, A549, H1229, and H1975) and bronchial epithelial (HBE) cells (Shanghai Institute of Biochemistry and Cell Biology, Shanghai, China) were cultured with DMEM containing 100 μg/ml streptomycin, 100 U/ml penicillin, and 10% FBS, in a 37℃ incubator with 5% CO_2_.

### 2.4 Bioinformatics analysis

The potential miRNA sponged by circWHSC1 and the potential downstream target gene of miR‐296‐3p were screened online (http://starbase.sysu.edu.cn).

### 2.5 Oligonucleotide interference and vector construction

The miRNA mimics were created by GenePharm (Shanghai, China). Small interfering RNAs (siRNAs) were created by Ribobio (Guangzhou, China). The luciferase reporters with circWHSC1 or AKT3 3′‐UTR sequence holding miR‐128‐3p binding sites were created by cloning the specific fragment into psiCHECK™‐2 vector. The point mutated reporters of circWHSC1 or AKT3 3′‐UTR were constructed after the conserved complementary nucleotides within miR‐128‐3p binding sites were mutated using a Gene Mutation Kit. The constructs were checked by sequencing and transfected into A549 and H1229 cells using Lipofectamine 2000.

### 2.6 RNA separation and qRT‐PCR assay

Total RNA was purified with Qiazol reagent, and cDNA was reverse transcribed using Revert Aid First Strand cDNA Synthesis Kit. RNA expression levels of target genes were measured by qRT‐PCR with SYBR Green PCR Master Mix on a Quantstudio™ DX Real‐Time PCR system (ABI), and normalized with U6 for miRNA and with GAPDH for circRNA. Primers were synthesized by GenePharm.

#### 2.6.1 Apoptosis and viability assays

Apoptosis was valued using Annexin V/FITC Apoptosis Detection Kit following manufacturer's guidebook and detected on a FACSCalibur Flow cytometer (BD.) using a CellQuest software (BDIS).

Cell viability was confirmed by CCK‐8 kit following manual from the manufacturer. Briefly, 10 μl of CCK‐8 reagent was added into each well of cells (3,000 cell/well/100 μl) in a 96‐well plate and cultured for 1 h at 37℃. Optional density (OD) was determined at 450 nm with a microplate reader.

### 2.7 Colony formation assessment

Cells (1,000/well) in 6‐well plates were routinely cultured for 7 days. Colonies were then fixed and stained, respectively, with 4% paraformaldehyde and 0.1% crystal violet solution. Colony images and numbers were collected.

### 2.8 Invasiveness and migration evaluates

Transwell chambers with polycarbonate filter (8.0‐μm pore size) were applied to carry out invasiveness (pre‐coated with Matrigel) and migration (without Matrigel) assays. For each well of the transwell chambers, 200 μl of serum‐free medium with 1 × 10^5^ cells were added into upper chamber, and 750 μl of 10% FBS containing medium was added into the lower chamber. After a 24 h (for migration assay) or 48 h (for invasiveness assay) incubation, the invaded and migrated cells were subjected to fix with 4% paraformaldehyde, stain with 0.1% crystal violet solution and photograph. Cell numbers were counted from 10 randomly selected fields of each chamber.

### 2.9 Western blot analysis

Cells in 10 cm cell culture dish were harvested with 500 μL RIPA lysis buffer, kept on ice for 20 min, centrifuged at 15,000 *g* and 4℃ for 15 min. Equal amount proteins (20 μg)were loaded on 8% SDS‐PAGE gel, transferred onto nitrocellulose membrane, blocked in 5% nonfat‐milk, hybridized respectively with different primary antibodies at 4℃ overnight and secondary antibodies at RT for 1 h. Then the target protein bands were visualized with SuperSignal West Dura Extended Duration Substrate.

### 2.10 Dual‐luciferase reporter assay

Being cultured in 24‐well plates (1 × 10^4^ cells/well) overnight, A549 and H1229 cells were subjected to co‐transfection of 20 nM miR‐296‐3p mimics (miR‐296‐3p) or negative control (miR‐NC) with 50 ng of psiCHECK‐2/circWHSC1 (WT) or psiCHECK‐2/circWHSC1 point mutated (MT) vector for circWHSC1 activity assay; while with 50 ng of psiCHECK‐2/AKT3 3′‐UTR (WT) or psiCHECK‐2/AKT3 3′‐UTR point mutated (MT) vector for AKT3 3′‐UTR activity assay, using Lipofectamine 2000, and then dual‐luciferase reporter assay kit was used to determine Firefly and Renilla luciferase activities following manufacturer's protocol.

### 2.11 RNA pull‐down assay

CircWHSC1 probes labeled with Biotin were synthesized in vitro by GenePharma, cultured with cell lysates and streptavidin agarose magnetic beads, and then eluted. Enriched miRNA was measured by qRT‐PCR.

### 2.12 Statistics analyses

Statistical significance was analyzed using SPSS 21.0 software (IBM), with *t* test for two group comparison, chi‐square test for multiple group comparison. Kaplan‐Meier method was applied for analyzing overall survival rate, and a Spearman correlation coefficient was applied for analyzing associations between gene expression levels. *p* value < 0.05 was statistically significant. *p < 0.05, **p < 0.01, ***p < 0.001.

## 3 RESULTS

### 3.1 CircWHSC1 plays an oncogenic role and serves as an indicator of poor prognosis in NSCLC patients

**FIGURE 1:**
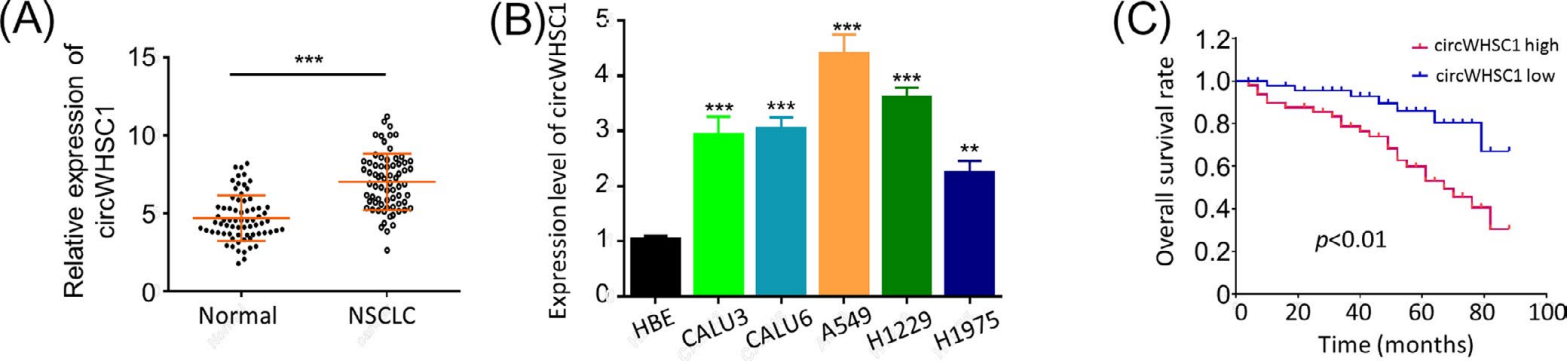
CircWHSC1 was up‐regulated in NSCLC cells and tissues, and an independent prognostic factor for overall survival of NSCLC patients. (A) qRT‐PCR assay showed a significantly higher circWHSC1 expression in NSCLC tissues versus the paired adjacent normal tissues (Normal) from 70 NSCLC patients; (B) relative circWHSC1 expression was significantly increased in the NSCLC cells (CALU3, CALU6, A549, H1229 and H1975) compared with the human bronchial epithelial (HBE) cells. Relative circWHSC1 expression was determined using the 2^−ΔΔCq^ method, and GAPDH was used as an internal control; (C) Kaplan‐Meier curve revealed a significantly decreased overall survival rates in NSCLC patients with high circWHSC1 expression. **p < 0.01;***p < 0.001

To reveal the fundamental function of circWHSC1 in NSCLC development, circWHSC1 expression profile in 70 NSCLC patient cancerous and self‐matched paracancerous tissues were investigated, which showed a significantly increased circWHSC1 expression level in the cancerous than in the paracancerous tissues (Figure 1A, *p* < 0.001). This finding was further confirmed by comparing circWHSC1 expressions between human NSCLC (CALU3, CALU6, A549, H1229, and H1975) and normal bronchial epithelial (HBE) cells. It can be seen in Figure 1B (*p* < 0.01 or *p* < 0.001), circWHSC1 expressions in tested NSCLC cells were all significantly up‐regulated versus the HBE, which were much higher in A549 and H1229 cells. To further reveal the clinical importance of circWHSC1 in NSCLC patients, we then analyzed the association between circWHSC1 expression levels with overall survival rate; the results confirmed that patients with high circWHSC1 expression level showed a significantly decreased overall survival rate (Figure 1C, *p* < 0.01). Therefore, these findings suggest that up‐regulation of circWHSC1 is significantly associated with poor prognosis of NSCLC patients.

### 3.2 CircWHSC1 promotes growth and metastasis of NSCLC cells in vitro

**FIGURE 2:**
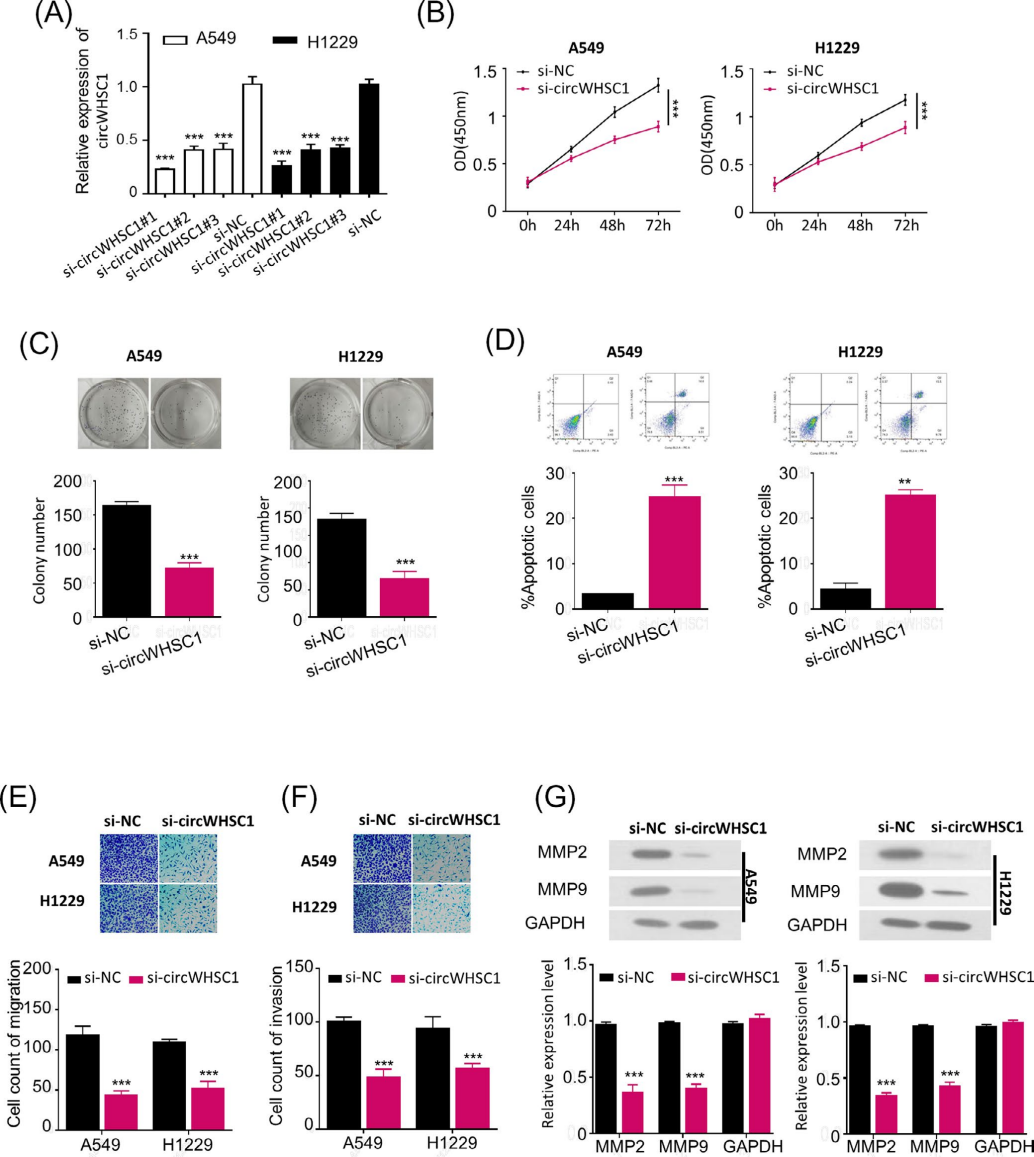
CircWHSC1 knockdown suppressed proliferation, invasion and migration, while induced apoptosis of NSCLC cells. (A) qRT‐PCR analysis confirmed the successful knockdown of circWHSC1 in A549 and H1229 cells respectively by transfection of si‐circWHSC1#1, si‐circWHSC1#2 or si‐circWHSC1#3, the following phenotypes were then assessed after transfection of si‐circWHSC1#1(named as si‐circWHSC1):(B)CCK‐8 assay analyzed cell viability; (C) colony formation assay analyzed colony numbers (left panel for images; right panel for quantitative analysis); (D) apoptosis detected by flow cytometric assay; transwell assay analyzed cell migration (E) and invasion (F) (upper panel for images; lower panel for quantitative analysis); (G) Western blotting assay determined protein expression levels of MMP2 and MMP9 (upper panel for images; lower panel for quantitative analysis). **p < 0.01;***p < 0.001

To evaluate the biological function of circWHSC1 during NSCLC oncogenesis and development, we first silenced circWHSC1 in A549 and H1229 cells, respectively, by transfection of three different siRNAs specifically targeting circWHSC1 (si‐circWHSC1#1, si‐circWHSC1#2, or si‐circWHSC1#3), which achieved an over 50% decrease of circWHSC1 expression in A549 and H1229 cells versus the negative control (si‐NC) (Figure 2A, *p* < 0.001), and si‐circWHSC1 #1 was most efficient. Therefore, si‐circWHSC1 #1 (designated as si‐circWHSC1) was used in the following experiments. Furthermore, we investigated the efficacy of circWHSC1 silence on the malignant characteristics of both A549 and H1229 cells, which showed that transfection of si‐circWHSC1 significantly inhibited cell viability time dependently (0–72 h, Figure 2B, *p* < 0.001), colony formation (Figure 2C, *p* < 0.001), migration (Figure 2E, *p* < 0.001), and invasion (Figure 2F, *p* < 0.001) abilities, as well as the expression levels of invasion‐associated proteins (MMP2 and MMP9)^23^ (Figure 2G, *p* < 0.001), while induced apoptosis (Figure 2D, *p* < 0.001 or *p* < 0.01), in both the A549 and H1229 cells versus the cells transfected with si‐NC. All these results point out that the oncogenic role of circWHSC1 in NSCLC is achieved by increasing colony formation ability, viability, migration, and invasion, while inhibiting apoptosis of NSCLC cells, which involves in the up‐regulation of MMP2 and MMP9 expression.

### 3.3 CircWHSC1 serves as a ceRNA of miR‐296‐3p in NSCLC cells

**FIGURE 3:**
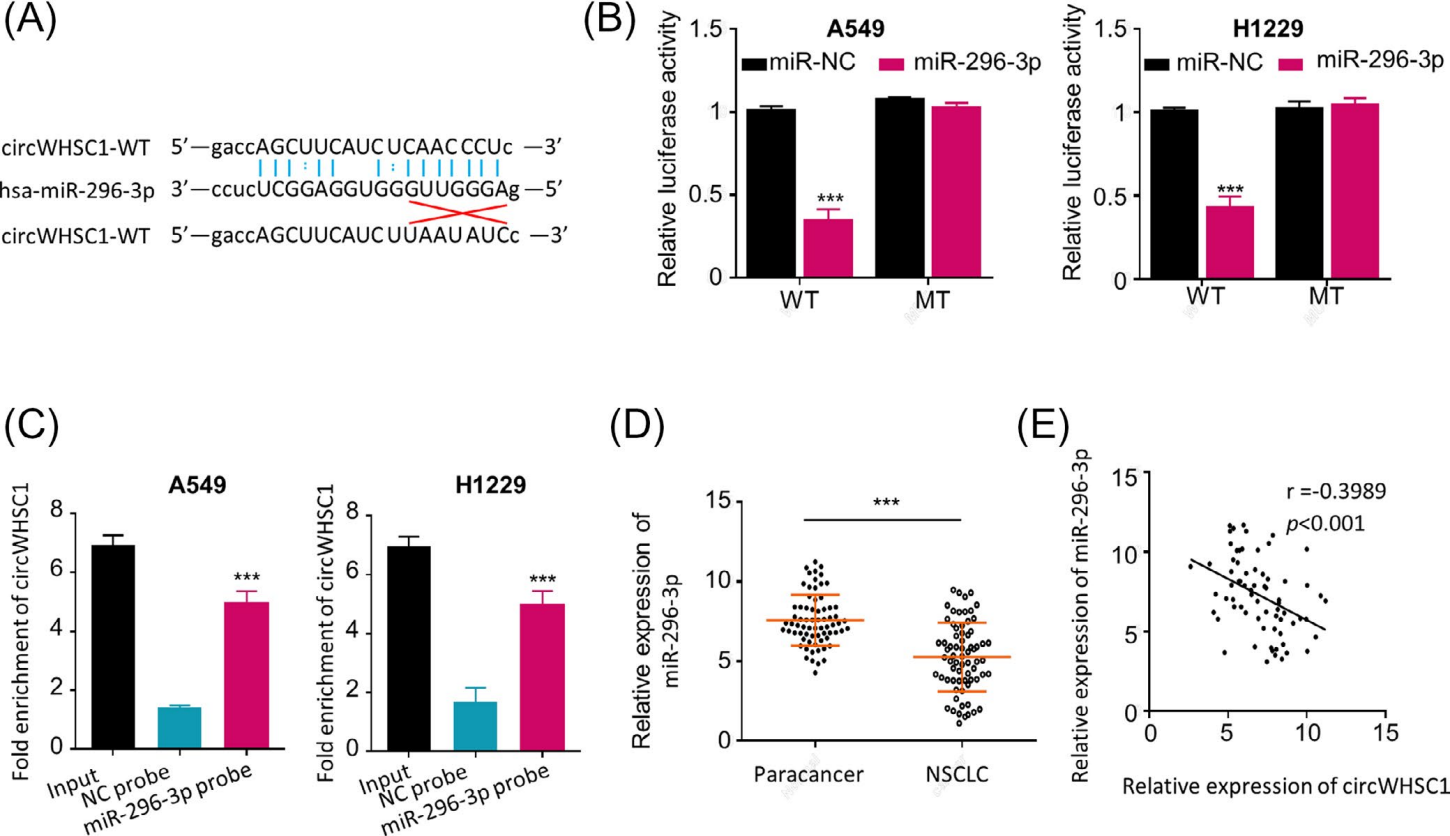
Exploring the prospective miRNA sponged by circWHSC1. (A) diagram of predicted (http://starbase.sysu.edu.cn) prospective binding sites of miR‐296‐3p with circWHSC1 and mutations used for specificity assay; (B) dual‐luciferase reporter assay in A549 and H1229 cells after co‐transfection of wild‐type (WT)/mutated (MT) circWHSC1 reporter gene together with miR‐296‐3p mimics/negative control (NC); (C) miR‐296‐3p in A549 and H1229 cells were pulled down and enriched by biotin‐labeled specific probe for circWHSC1; (D) qRT‐PCR assay showed a significantly lower miR‐296‐3p expression in NSCLC tissues versus the paired adjacent normal tissues (Normal) from 70 NSCLC patients; (E) correlation between circWHSC1 and miR‐296‐3p expression levels in 70 NSCLC tissues by Spearman's correlation coefficient analysis. Relative miR‐296‐3p levels were determined by 2^−ΔΔCq^ method with U6 as the internal control. **p < 0.01;***p < 0.001

Studies have shown that circRNAs predominantly act as ceRNAs of miRNAs to regulate their target gene expression,^24^, ^25^ we therefore investigated the potential miRNA that is sponged by circWHSC1 in NSCLC cells. MiR‐296‐3p,^21^, ^22^ a miRNA suppressing viability, migration and invasiveness of NSCLC cells, was identified as a potential target of circWHSC1 by on line bioinformatics analysis (http://starbase.sysu.edu.cn) (Figure 3A). RNA pull‐down and dual‐luciferase reporter assay were performed in A549 and H1229 cells to further confirm the direct binding between miR‐296‐3p and circWHSC1 in NSCLC cells. As we can see in Figure 3B, over 50% reporter activity was inhibited in the presence of wild‐type circWHSC1 reporter gene (WT), however, no significant inhibition was found in the presence of circWHSC1‐mut (MT); meanwhile, more miR‐296‐3p was pulled down by circWHSC1 probe than the negative control oligo probe (NC probe) in both A549 and H1229 cells (Figure 3C, *p* < 0.001), indicating that circWHSC1 can directly bind to miR‐296‐3p in NSCLC cells. Moreover, we confirmed that miR‐296‐3p expression was significantly lower in 70 NSCLC patient cancerous tissues versus the paired paracancerous tissues (Figure 3D, *p <* 0.001), and the Spearman correlation coefficient analysis showed that circWHSC1 expression level was significantly negative correlated with miR‐296‐3p expression level in the 70 NSCLC tissues (Figure 3E, *p *< 0.001). Therefore, the fundamental mechanism of circWHSC1 in NSCLC oncogenesis is to serve as a sponge of miR‐296‐3p.

### 3.4 CircWHSC1 promotes NSCLC oncogenesis and progression via sponging miR‐296‐3p

**FIGURE 4:**
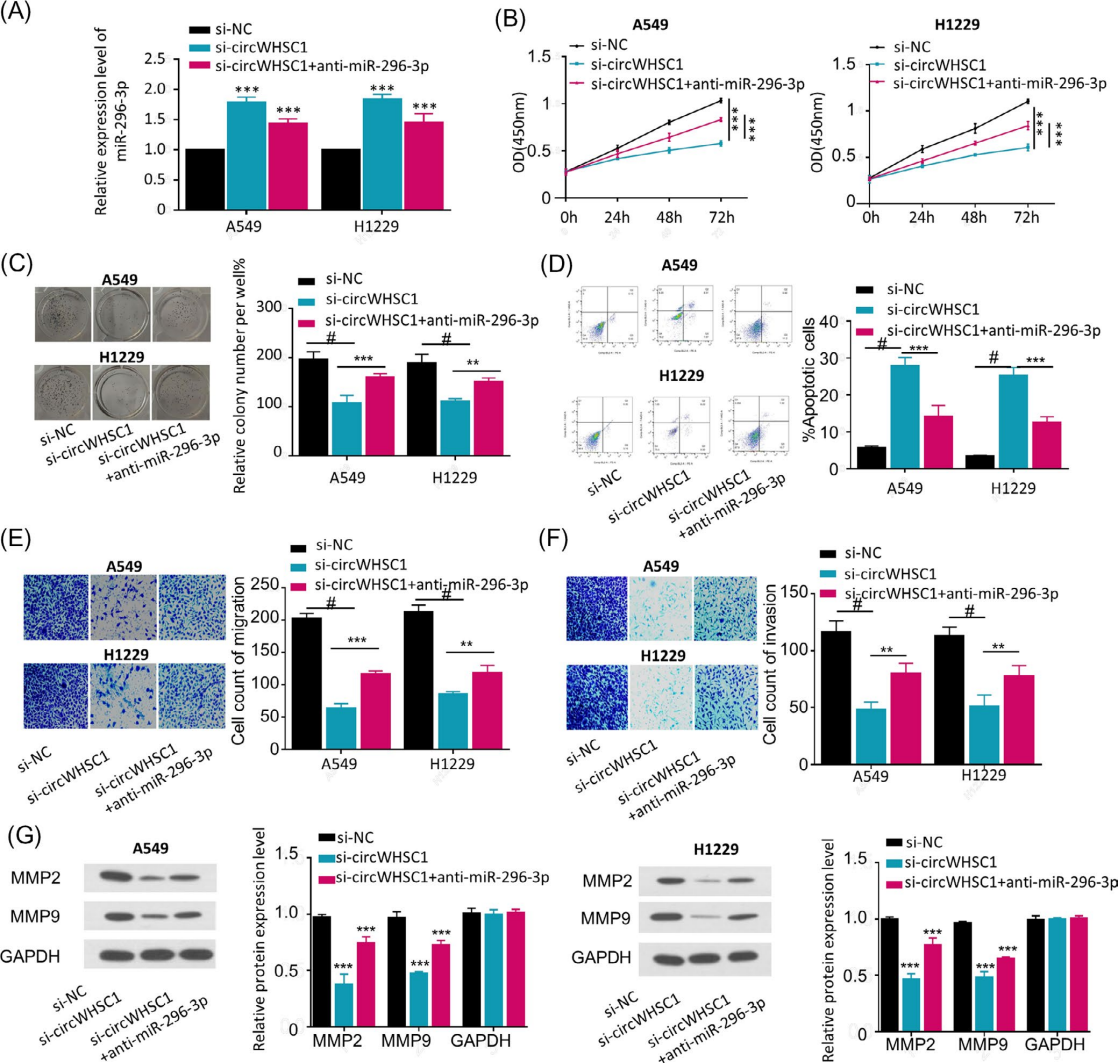
CircWHSC1 stimulated NSCLC development by sponging miR‐296‐3p expression. CircWHSC1 was silenced without or with anti‐miR‐296‐3p (si‐NC, si‐circWHSC1, si‐circWHSC1+anti‐miR‐296‐3p)in A549 and H1229 cells, then the following parameters were checked, (A) miR‐296‐3p expression levels by qRT‐PCR; (B) viability by CCK‐8 assay; (C) colony formation ability; (D) apoptosis by flow cytometric assay; (E) migration by transwell assay; (F) invasion by transwell assay; (G) protein expression levels of MMP2 and MMP9 by Western blotting. **p < 0.01;***p < 0.001

To understand whether circWHSC1 plays its critical role in promoting NSCLC oncogenesis and progression via sponging miR‐296‐3p, we performed rescue experiments by transfection of si‐NC, si‐circWHSC1 or si‐circWHSC1+anti‐miR‐296‐3p in A549 and H1229 cells (Figure 4A, *p* < 0.001), followed by cell function assays, including cell viability, colony formation ability, apoptosis, invasiveness, and migration abilities. The results demonstrated that circWHSC1 silence (si‐circWHSC1) decreased the cell viability (Figure 4B, *p* < 0.001), the colony formation ability (Figure 4C, *p* < 0.001), the migration ability (Figure 4E, *p *< 0.01), the invasiveness ability (Figure 4F, *p* < 0.01), and protein expression levels of MMP2 and MMP9 (Figure 4G, *p* < 0.01), while induced apoptosis (Figure 4D, *p* < 0.01) in both A549 and H1229 cells; while inhibition of miR‐296‐3p by co‐transfecting miR‐296‐3p inhibitor (si‐circWHSC1+anti‐miR‐296‐3p) partially restored the proliferative and invasive characteristics of A549 and H1229 cells that were inhibited by circWHSC1 silence (Figure 4B–G). These results suggest that circWHSC1 promotes growth and invasion of NSCLC cells by sponging miR‐296‐3p.

### 3.5 CircWHSC1 up‐regulates AKT3 via sponging miR‐128‐3p to release inhibitory effect of miR‐128‐3p on AKT3

**FIGURE 5:**
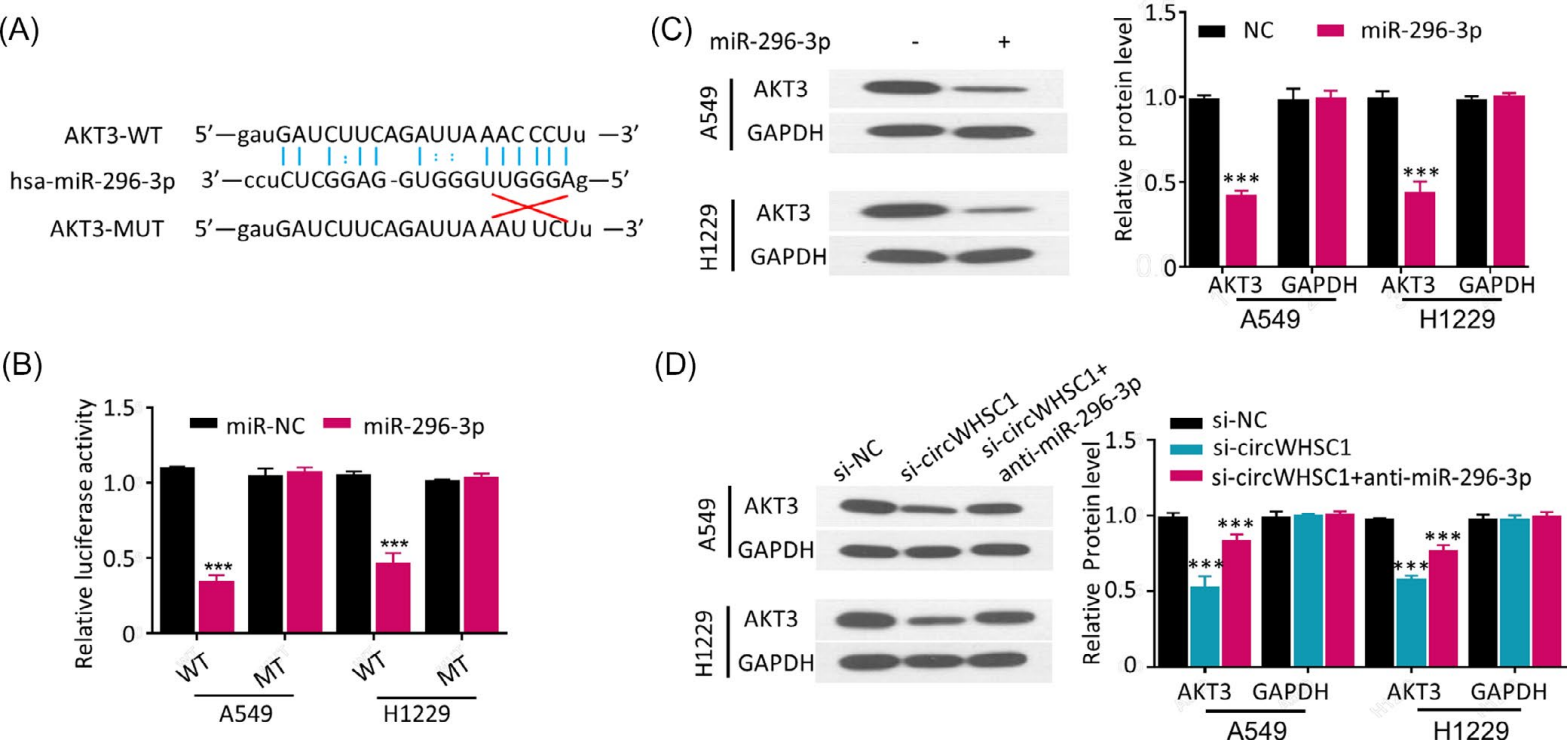
CircWHSC1 up‐regulated AKT3 expression in NSCLC cells by sponging miR‐296‐3p. (A) Diagram of predicted prospective binding sites between miR‐296‐3p and AKT3 (http://starbase.sysu.edu.cn), and mutations used for specificity confirmation;(B)dual‐luciferase reporter analysis in A549 and H1229 cells after co‐transfection of wild‐type (WT)/mutated (MT) AKT3 3′‐UTR reporter gene with miR‐296‐3p mimics/negative control (NC); (C) Western blotting assay detected AKT3 protein expression in A549 and H1229 cells after miR‐296‐3p over‐expression (left panel, images; right panel, quantitative analysis); (D) Western blotting assay detected AKT3 protein expression in Figure 1 A549 and H1229 cells after circWHSC1 knockdown without or with anti‐miR‐296‐3p (si‐NC, si‐circWHSC1, si‐circWHSC1+anti‐miR‐296‐3p; left panel, images; right panel, quantitative analysis)

The results above showed that circWHSC1 served as a ceRNA of miR‐296‐3p to promote growth and invasion of NSCLC cells, to further explore whether circWHSC1 could increase expression of the potential target gene that directly interacted with miR‐296‐3p in NSCLC cells, we then further performed the online bioinformatics analysis to detect the possible mRNA holding complementary sequence in the 3′‐UTR with miR‐296‐3p (http://starbase.sysu.edu.cn). As we can see in Figure 5A, a miR‐296‐3p binding site on the AKT3 3′‐UTR was discovered. Moreover, direct interaction between miR‐296‐3p and AKT3 3′‐UTR in A549 and H1229 cells was confirmed by dual‐luciferase reporter assay. As a result, over‐expression of miR‐296‐3p with mimics (miR‐296‐3p) decreased the luciferase activities of A549 and H1229 cells in the presence of wild‐type AKT3 3′‐UTR reporter (WT) (Figure 5B, *p* < 0.001), however, over‐expression of miR‐296‐3p did not decrease the luciferase activities of A549 and H1229 cells in the presence of mutated AKT3 3′‐UTR reporter (MT) (Figure 5B). To determine whether circWHSC1 sponged miR‐296‐3p regulates AKT3 function, we first checked AKT3 expression in miR‐296‐3p over‐expressed NSCLC cells. The data showed that miR‐296‐3p over‐expression considerably down‐regulated AKT3 expression levels of A549 and H1229 cells (Figure 5C, *p* < 0.001); furthermore, AKT3 expression was checked in circWHSC1 silenced NSCLC cells, which showed that circWHSC1 silence caused a significant down‐regulation of AKT3 protein expression in A549 and H1229 cells, while AKT3 protein expression was almost rescued after co‐transfecting anti‐miR‐296‐3p (Figure 5D, *p *< 0.001). These results reveal that circWHSC1 promotes AKT3 expression in NSCLC cells by blocking the inhibitory effects of miR‐296‐3p.

## 4 DISCUSSION

Benefits from rapid development of high‐throughput and bioinformatics technologies, the biological functions of circRNAs in oncogenesis and cancer development have attracted great attentions in the biomedical research field, as a result, some circRNAs have been revealed to be the potential diagnostic, therapeutic and prognostic targets in NSCLC.^26^, ^27^, ^28^ It has been reported that circ‐SMARCA5 is a tumor suppressor in NSCLC^26^; hsa_circ_0007385 is a new biomarker for monitoring disease and predicting prognosis in NSCLC patients ^27^; circRNA_103762 stimulates multidrug resistance in NSCLC via targeting DNA damage‐inducible transcript 3 (CHOP).^28^ However, only few circRNAs have been well investigated, the biological functions and molecular mechanisms of majority circRNAs in NSCLC need to be further explored.

Here, we compared the expression profile of circWHSC1 between the cancerous and paracancerous tissues of 70 NSCLC patients, as well as the five different human NSCLC and the normal bronchial epithelial (HBE) cells; furthermore, the correlation between circWHSC1 expression levels and overall survival rate of NSCLC patients was analyzed using Kaplan‐Meier method. As a result, we revealed a significantly increased circWHSC1 expression in the cancerous tissues of NSCLC patients; the up‐regulated circWHSC1 was further confirmed in the tested NSCLC cells, suggesting the potential carcinogenesis role of circWHSC1 in NSCLC. Meanwhile, we discovered the significant association between high circWHSC1 expression level with decreased overall survival rate of NSCLC patients, leading to a poor prognosis. This highlighted the possibility of circWHSC1 as a potential prognostic biomarker in NSCLC patients.

CircRNAs have been well known to act as ceRNAs of miRNAs.^29^ To explore whether circWHSC1 also functions as a ceRNA in promoting NSCLC development, bioinformatics analysis was first performed to predict the potential binding miRNAs, and miR‐296‐3p, a tumor‐suppressor miRNA in NSCLC, was recognized; second, dual‐luciferase reporter assay and circRNA/miRNA pull‐down analysis both provided evidences of direct binding between circWHSC1 and miR‐296‐3p. Finally, anti‐miR‐296‐3p treatment partially reversed circWHSC1 silence induced decrease of cell proliferation and metastasis in A549 and H1229 cells, indicating the carcinogenestic effect caused by up‐regulated circWHSC1 could be reversed by miR‐296‐3p mimics, which could be a novel therapeutic strategy for NSCLC patients.

Since the ceRNA principle believes that RNA transcripts, such as circRNAs, regulate each other's expression via competing their shared miRNA response elements (MREs).^30^ Here, we identified that circWHSC1 shared same MRE of miR‐149‐5p with AKT3 after bioinformatics analyses. It has been reported that AKT3 is aberrantly expressed in indifferent type of cancers, such as NSCLC, indicating the importance of AKT3 in regulating NSCLC development.^31^ Therefore, the direct interaction between miR‐149‐5p and AKT3 was further confirmed with the dual‐luciferase reporter assay in the current study; meanwhile, miR‐296‐3p over‐expression was found to significantly down‐regulate AKT3 protein expression in A549 and H1229 cells; furthermore, circWHSC1 silence significantly down‐regulated AKT3 protein expression in A549 and H1229 cells, which was partially rescued in the presence of anti‐miR‐296‐3p. Therefore, these findings provide experimental evidences for the new mechanism that circWHSC1 serves as a ceRNA of miR‐296‐3p to increase AKT3 expression in promoting NSCLC development.

## 5 CONCLUSIONS

Our study first revealed that circWHSC1 was significantly increased in both NSCLC patient tissues and cells, and an independent predictor for poor prognosis of NSCLC patients. CircWHSC1 promoted colony formation, viability, invasiveness, and migration, while inhibiting apoptosis of NSCLC cells by up‐regulating AKT3 expression after sponging miR‐296‐3p, therefore, contributed to NSCLC metastasis. These discoveries highlight a prospective function of circWHSC1 in promoting NSCLC development; therefore, targeting circWHSC1 may provide novel strategies for diagnosis, therapeutics, and prognosis of NSCLC management.

## CONFLICT OF INTEREST

No competing interests.

## ETHICS APPROVAL AND INFORMED CONSENT

All samples were obtained with informed consents from patients, and the experimental processes were approved by our Hospital's Ethics Committee.

## Data Availability

Data supporting this work is available from the corresponding author per request.
